# A Matching-Adjusted Indirect Comparison of Pembrolizumab + Chemotherapy vs. Nivolumab + Ipilimumab as First-Line Therapies in Patients with PD-L1 TPS ≥1% Metastatic NSCLC

**DOI:** 10.3390/cancers12123648

**Published:** 2020-12-04

**Authors:** Balazs Halmos, Thomas Burke, Chrysostomos Kalyvas, Ralph Insinga, Kristel Vandormael, Andrew Frederickson, Bilal Piperdi

**Affiliations:** 1 Department of Oncology, Montefiore/Albert Einstein Cancer Center, 2nd floor, 1695 Eastchester Rd, Bronx, NY 10461, USA; 2 Center for Observational & Real World Evidence (CORE), Merck & Co., Inc., Kenilworth, NJ 07033, USA; thomas_burke2@merck.com (T.B.); ralph_insinga@merck.com (R.I.); 3 Biostatistics and Research Decision Sciences, MSD Europe, Inc., 1200 Brussels, Belgium; chrysostomos.kalyvas@merck.com (C.K.); kristel_vandormael@merck.com (K.V.); 4 Precision HEOR, Oakland, CA 94612, USA; andrew.frederickson@precisionvh.com; 5 Oncology Clinical Development, Merck & Co., Inc., Kenilworth, NJ 07033, USA; bilal.piperdi@merck.com

**Keywords:** non-small cell lung cancer, matching-adjusted indirect comparison, comparative effectiveness, pembrolizumab, nivolumab, chemotherapy

## Abstract

**Simple Summary:**

Pembrolizumab + chemotherapy and nivolumab + ipilimumab are approved first-line treatment options for patients with metastatic stage IV NSCLC with a PD-L1 tumor proportion score (TPS) ≥1%. Understanding the differences between these treatment options is required to inform clinical decision making. In the absence of head-to-head trials, this study indirectly compared the effectiveness of pembrolizumab + chemotherapy vs nivolumab + ipilimumab in this patient population by performing a matching-adjusted indirect comparison. Pembrolizumab + chemotherapy demonstrated clinical benefit compared to nivolumab + ipilimumab in patients with PD-L1 TPS ≥1% across multiple endpoints. Sub-group analyses in PD-L1 TPS ≥50% and 1–49% patients were consistent with results in the overall PD‑L1 TPS ≥1% population. This study provides important results to inform clinical decision making given the lack of head-to-head studies comparing pembrolizumab + chemotherapy and nivolumab + ipilimumab.

**Abstract:**

Background: In the absence of head-to-head trials, this study indirectly compared the effectiveness of pembrolizumab + chemotherapy vs nivolumab + ipilimumab for the first-line treatment of metastatic stage IV NSCLC patients with PD-L1 tumor proportion score (TPS) ≥1%. Methods: An anchored matching-adjusted indirect comparison (MAIC) was conducted using pooled individual patient data (IPD) from the ITT population in KEYNOTE-021G, KEYNOTE-189 and KEYNOTE-407 (*n* = 816) and published aggregate data of nivolumab + ipilimumab from CheckMate 227 Part 1A (*n* = 793). To adjust for cross-trial differences in baseline characteristics, data from KEYNOTE-021G/KEYNOTE-189/KEYNOTE-407 were re-weighted to match the baseline characteristics of CheckMate 227 Part 1A. Outcomes included OS, PFS and ORR. Base case analyses were restricted to patients with PD-L1 TPS ≥1%, with sub-group analyses in PD-L1 TPS ≥50% and 1–49%. Results: The estimated HR (95% CI) of pembrolizumab + chemotherapy vs nivolumab + ipilimumab was 0.80 (0.59,1.09) and 0.53 (0.41,0.68) for OS and PFS, respectively. For ORR, the estimated risk ratio was 1.8 (1.3,2.4) for pembrolizumab + chemotherapy vs nivolumab + ipilimumab and the risk difference was 25.5% (15.0,36.0). PD-L1 TPS ≥50% and 1–49% sub-groups showed an OS HR of 0.89 (0.58,1.36) and 0.68 (0.46,1.01), respectively. Conclusion: These MAIC results suggest that pembrolizumab + chemotherapy leads to a greater clinical benefit vs nivolumab + ipilimumab in patients with PD-L1 TPS ≥1% across multiple endpoints.

## 1. Introduction

With the introduction of immunotherapy, the availability of therapeutic options in metastatic NSCLC has rapidly expanded, including programmed cell death protein-1 (PD 1)/programmed death-ligand 1 (PD-L1) immune checkpoint inhibitors [1,2].

Pembrolizumab + platinum chemotherapy is an approved treatment for previously untreated metastatic NSCLC patients, irrespective of tumor PD-L1 expression [3,4]. In the phase II KEYNOTE-021 Cohort G (KN021G) trial of patients with advanced non squamous NSCLC without epidermal growth factor receptor (EGFR) or anaplastic lymphoma kinase (ALK) aberrations, the hazard ratio (HR) for overall survival (OS) with pembrolizumab + carboplatin + pemetrexed vs with pemetrexed + carboplatin alone was 0.56 (95% confidence interval [CI] 0.32‒0.95; *p* value = 0.015) after 24 months of follow-up [5]. Progression-free survival (PFS) was also significantly improved in the pembrolizumab + carboplatin + pemetrexed arm compared to pemetrexed + carboplatin alone (HR = 0.53; 95% CI 0.33‒0.86; *p* value = 0.005) [5]. In the phase III KEYNOTE-189 (KN189) trial, pembrolizumab + pemetrexed + platinum chemotherapy resulted in a significantly improved OS (HR = 0.49; 95% CI 0.38‒0.64; *p* value < 0.001) and PFS (HR = 0.52; 95% CI 0.43‒0.64; *p* value < 0.001) compared to placebo + pemetrexed + platinum chemotherapy [6]. Furthermore, in the phase III KEYNOTE-407 (KN407) trial of patients with previously untreated metastatic squamous NSCLC, the addition of pembrolizumab to carboplatin plus paclitaxel or nab-paclitaxel resulted in significantly longer OS (HR = 0.64; 95% CI 0.49‒0.85; *p* value < 0.001) and PFS (HR = 0.56; 95% CI 0.45‒0.70; *p* value < 0.001) than chemotherapy alone [7].

Nivolumab + ipilimumab received United States Food and Drug Administration (FDA) regulatory approval in May 2020 for previously untreated metastatic NSCLC with PD-L1 TPS ≥1% and no EGFR/ALK aberrations [3,8]. In CheckMate 227 Part 1A, the nivolumab + ipilimumab arm was associated with a significantly longer OS than platinum-based chemotherapy in metastatic NSCLC patients with PD-L1 TPS ≥1% (HR = 0.79; 97.72% CI [chosen based on hierarchical testing procedure] 0.65–0.96) [9].

As there are multiple FDA-approved anti-PD-1/PD-L1 first-line treatment options available, a comprehensive understanding of the potential differences between these options is required to inform clinical decision making. Treatment choices are often determined based on the evaluation of survival outcomes, alongside balancing toxicity concerns, treatment duration and cost considerations. The benefits of immunotherapy are also dependent on biomarker selection, including the status of PD-L1 expression [10]. Pembrolizumab + chemotherapy and nivolumab + ipilimumab have both demonstrated benefit in their respective clinical trials compared with platinum doublet chemotherapy. However, no direct head-to-head randomized controlled trials (RCTs) have compared these regulatory-approved regimens given the large patient sample size and time-frame required to obtain results.

Given the paucity of head-to-head trials in this population, indirect treatment comparison (ITC) is a useful approach to evaluate the comparative effectiveness of first-line regimens from different trials and provide useful insights for clinicians, patients and policy makers to make an informed selection between different FDA-approved treatment options. Matching-adjusted indirect comparison (MAIC) is becoming increasingly prominent and accepted by health technology assessment agencies as a robust statistical approach to provide comparative effectiveness evidence and inform clinical decision making [11,12,13,14,15].

The objective of this study was to assess the comparative effectiveness of two FDA-approved treatment regimens, pembrolizumab + chemotherapy vs nivolumab + ipilimumab, for first-line treatment in patients with NSCLC with PD-L1 TPS ≥1% by employing a MAIC.

## 2. Materials and Methods

MAIC statistically controls for cross-trial differences in observed relative treatment effect modifiers rather than utilizing the assumption that effect modifiers are balanced across trials. MAIC is appropriate when individual patient data (IPD) from one trial and aggregate data from the second trial are available. In this study, IPD from pooled KN021G, KN189 and KN407 trials, and aggregate data reported for CheckMate 227 Part 1A were used to perform a MAIC of pembrolizumab + chemotherapy vs nivolumab + ipilimumab using the common comparator (platinum doublet chemotherapy) as the anchor [5,6,7,9,16].

### 2.1. Study Populations

Four studies were identified that evaluated the interventions of interest for the first-line treatment of metastatic NSCLC patients with PD-L1 TPS ≥1%, as detailed below. The patient enrollment criteria between the selected studies were similar, including patients aged ≥18 years, with Eastern Cooperative Oncology Group (ECOG) performance score ≤1, and stage IV disease whilst KN021G also included stage IIIb disease. Study characteristics are presented in Table 1. The four studies included here were also identified as part of a systematic literature review that was conducted to investigate the efficacy of PD-1/PD-L1 inhibitors in the first-line advanced NSCLC setting [17].

KN021G (NCT02039674; database cut-off date: 19 August 2019) was a randomized, open label, phase II trial of pembrolizumab + carboplatin + pemetrexed vs carboplatin + pemetrexed in previously untreated stage IIIB/IV non-squamous NSCLC patients without EGFR/ALK aberrations [5]. KN189 (NCT02578680; database cut-off date: 20 May 2019) was a randomized, double-blind, phase III trial of pembrolizumab + pemetrexed + platinum chemotherapy vs saline placebo + pemetrexed + platinum chemotherapy in previously untreated stage IV non-squamous NSCLC patients without EGFR/ALK aberrations [6,16]. KN407 (NCT02775435; database cut-off date: 9 May 2019) was a randomized, double blind, phase III trial of pembrolizumab + carboplatin + paclitaxel/nab-paclitaxel vs saline placebo + carboplatin + paclitaxel/nab-paclitaxel in previously untreated stage IV squamous NSCLC patients [7]. KN407 did not have an explicit inclusion/exclusion criterion around EGFR/ALK status, as KN407 was restricted to squamous patients, a population with a very low prevalence of EGFR/ALK mutation. Within trial cross-over to pembrolizumab monotherapy was permitted among patients randomized to the chemotherapy control arm following verified disease progression in KN021G, KN189 and KN407. At the time of data cut-off for the OS analysis used here, the percentage of patients who received pembrolizumab or PD 1/PD L1 directed therapy in the control arm (within trial and outside of trial cross-over) was 68.3% for KN021G and 54.9% for KN189 and 49.1% for KN407 [5,6,7,16].

CheckMate 227 Part 1A (NCT02477826; database cut-off date 2 July 2019) was a randomized, open label, phase III trial of nivolumab + ipilimumab or nivolumab monotherapy vs platinum doublet chemotherapy among previously untreated stage IV NSCLC patients with PD-L1 TPS ≥1% and no EGFR/ALK tumor aberrations [9]. This study focused on the FDA-approved indication for nivolumab + ipilimumab, therefore nivolumab monotherapy was not examined in this MAIC. The study protocol for CheckMate 227 Part 1 originally specified OS as the primary endpoint in patients with PD-L1 TPS ≥1% but was later amended to include the co primary endpoint of PFS in patients with high tumor mutational burden [9]. To ensure a representative population for the MAIC, the outcomes for nivolumab + ipilimumab and chemotherapy arms were derived from patients with PD-L1 TPS ≥1%, as originally assessed and presented in the CheckMate 227 Part 1A publication. Within trial cross-over between the treatment arms during the CheckMate 227 Part 1A trial was not permitted but could occur upon withdrawal [9]. At the time of data cut-off for the OS analysis used here, the percentage of patients who received PD 1/PD L1 directed therapy in the control arm (outside of trial cross over) was 43.1% for CheckMate 227 Part 1A [9].

### 2.2. Outcome Measures

Outcomes consisted of OS, blinded independent reviewer-assessed PFS and objective response rate (ORR), with similar clinical endpoint definitions across trials. In KN021G/KN189/KN407 and CheckMate 227 Part 1A, OS was defined as the time from randomization to death due to any cause; PFS was defined as the time from randomization to the first documented disease progression per Response Evaluation Criteria in Solid Tumors (RECIST) 1.1 based on independent review or death due to any cause, whichever occurred first, expressed in days; and ORR was defined as the proportion of patients who have a complete response or a partial response. Patients without documented death were censored at the day of the last contact for OS, while patients without an event (progression or death) at the time of last tumor assessment were censored at the last disease assessment date for PFS.

IPD were sourced for the pembrolizumab + chemotherapy and chemotherapy arms from the KN021G, KN189 and KN407 trials. The outcomes of interest were extracted as HRs and 95% CIs for OS and PFS and as proportions for ORR from the CheckMate 227 Part 1A trial publication [9]. For KN021G, KN189 and KN407, the outcome (OS, PFS, ORR) in each treatment group was compared within each study (strata) by the log rank and the differences within each stratum were then combined to give an overall comparison of treatments that has been adjusted for the study. The Kaplan–Meier curves from the CheckMate 227 Part 1A trial publication were also digitized using DigitizeIt v2.3. Estimated patient-level data (pseudo-IPD) for the CheckMate 227 Part 1A trial were then derived employing the method developed by Guyot et al., 2012 [18] using the number of patients at risk over time alongside digitized Kaplan–Meier curves.

### 2.3. Statistic Methods

The first step when implementing a MAIC is to align relevant inclusion and exclusion criteria of the studies by removing patients from the dataset with full IPD that could not have been enrolled in the comparator study. Data from KN021G, KN189 and KN407 were pooled to increase the potential effective sample size (ESS) of the pembrolizumab + chemotherapy and chemotherapy arms. ESS is the number of independent non-weighted individuals required to give an estimate with the same precision as the weighted sample estimate.

Some differences in the stage and occurrence of disease were observed in the inclusion criteria. Checkmate 227 Part 1A included patients with stage IV or recurrent NSCLC. Conversely, KN021G included patients with stage IIIB or IV while KN189 and KN407 included patients with stage IV NSCLC. The discrepancy regarding the stage of the disease was adjusted by restricting to patients with stage IV disease only. However, the discrepancy regarding patients with recurrent NSCLC was not adjusted for, given that the KEYNOTE studies did not collect data on recurrence status of stage IV patients and Checkmate 227 Part 1A did not report the distribution of recurrent stage IV patients within the publication. Furthermore, the follow-up was different between trials, as pooled KN021G/KN189/KN407 and CheckMate 227 Part 1A had a maximum patient follow-up of 50 months and 42 months, respectively. In the base case analysis, to adjust for differences in follow-up for OS and PFS, data from KN021G/KN189/KN407 were truncated to the end of follow-up in CheckMate 227 Part 1A by censoring patient outcomes reported after this date.

Patients across KN021G/KN189/KN407 and CheckMate 227 Part 1A trials were matched on a range of potential effect modifiers, including age, sex, geographic region, ECOG performance status, smoking status, histology, sites of metastases, and PD-L1 expression, in line with published guidance from the National Institute for Health and Care Excellence (NICE) Decision Support Unit [15]. The selection of potential effect modifiers was based on a statistical assessment of the baseline characteristics reported in trials, clinical expertise and review of the clinical literature [19,20,21,22].

Analyses were restricted to the patient population with PD-L1 TPS ≥1%. Data from patients receiving pembrolizumab + chemotherapy and chemotherapy were re-weighted to match the baseline characteristics of patients included in CheckMate 227 Part 1A receiving nivolumab + ipilimumab and chemotherapy. The individual weights were estimated using a logistic model as described by Signorovitch et al., 2010 [23].

OS and PFS analyses were conducted using two approaches: the primary approach used the aggregate data while the secondary approach used pseudo-IPD from CheckMate 227 Part 1A. For the primary approach, the results (estimate of treatment effects, log(HR) and standard errors) of the weighted Cox model from KN021G/KN189/KN407 were used together with the aggregate results reported in CheckMate 227 Part 1A. The ITC based on Bucher et al., 1997 was then performed to obtain the (after matching) ITC HR and its 95% CIs [24]. For the secondary approach, a Cox proportional hazards model on the pseudo-IPD from Checkmate 227 Part 1A and weighted IPD from KN021G/KN189/KN407 was used to determine the treatment effect of pembrolizumab + chemotherapy relative to nivolumab + ipilimumab. To test the impact of the truncated data that were adjusted for the differences in follow-up on the outcomes and assess the robustness of the MAIC analyses, additional analyses were conducted for the OS and PFS endpoints using complete follow-up from each study.

All analyses were conducted using SAS 9.4. The standard errors for MAIC estimates were calculated using a robust sandwich estimator derived empirically from the data and accounted for the fact that the weights were estimated rather than being fixed and known [23]. The ESS, as defined in Signorovitch et al., 2010 [23], is derived from the estimates using linear combinations of the observations. When using weighted survival estimates, ESS cannot be easily calculated, as the survival estimates are not a linear function of the observations. Therefore, the number of patients at risk, computed as the sum of the weights, differs from the ESS presented in the table with the baseline characteristics.

Sub-group analyses were conducted for all outcomes for patients with PD-L1 TPS 1–49% and ≥50% as it is recognized that PD-L1 expression may affect treatment effectiveness. Summary baseline characteristics of patients with PD-L1 TPS 1–49% and ≥50% in CheckMate 227 Part 1A were not reported in Hellmann et al., 2019. Baseline characteristics were generally evenly distributed in patients with PD-L1 TPS 1–49% vs those with PD-L1 TPS ≥50% in KN407 and KN189 (KN021G was not examined due to the small sample size) [6,7,16]. Therefore, it was assumed that baseline characteristics, except PD-L1 status, followed the same distribution as the overall PD-L1 TPS ≥1% population. The treatment effect of pembrolizumab + chemotherapy vs nivolumab + ipilimumab was estimated in patients with PD-L1 TPS 1–49% using the primary approach since the aggregate data for nivolumab + ipilimumab in this sub-group population were reported in the CheckMate 227 Part 1A publication. However, the secondary approach was not applied for the PD-L1 TPS 1–49% sub-group, as the Kaplan–Meier plots for the sub-group population were not available in the CheckMate 227 Part 1A publication; therefore, a pseudo-IPD could not be estimated for this population [9].

## 3. Results

### 3.1. Baseline Characteristics

Before matching, a few differences in baseline characteristics between the two populations were observed. Patients randomized to pembrolizumab + chemotherapy and chemotherapy arms from KN021G/KN189/KN407 included a lower proportion of patients with non-squamous NSCLC (56.74% vs 70.62%) and a greater proportion of patients with squamous NSCLC (43.26% vs 29.38%) than those randomized to nivolumab + ipilimumab and chemotherapy arms from CheckMate 227 Part 1A. Patients randomized to pembrolizumab + chemotherapy and chemotherapy arms from KN021G/KN189/KN407 also had a higher proportion of patients from North America (25.86% vs 11.98%) and a lower proportion of patients from Asia (8.46% vs 20.43%) than those randomized to nivolumab + ipilimumab and chemotherapy arms from CheckMate 227 Part 1A. Other baseline factors were well-balanced between the two populations. The distribution of baseline characteristics in Table 2 was identical after matching. The differences in the baseline characteristics between studies were a contributory factor to the ESS of the pembrolizumab + chemotherapy and chemotherapy arms being smaller (456) than the sample size before matching (816). A 44% reduction from the original sample size was observed, suggesting a reasonably high degree of similarity between the patient populations compared prior to matching.

### 3.2. Base Case Analysis

Table 3 presents the OS and PFS results in NSCLC patients with PD-L1 TPS ≥1% after matching (see Table S1 for results using populations before matching). The estimated treatment effect on both OS and PFS appeared more favorable towards pembrolizumab + chemotherapy compared with nivolumab + ipilimumab.

For OS, the estimated HR after matching based on the primary approach of pembrolizumab + chemotherapy vs nivolumab + ipilimumab was 0.80, similar to the before matching result which was 0.84. Based on the secondary approach, the estimated HR for OS with pembrolizumab + chemotherapy vs nivolumab + ipilimumab was 0.75 after matching, similar to the before matching result, which was 0.82. The median OS was higher among patients who received pembrolizumab + chemotherapy compared to those who received nivolumab + ipilimumab, regardless of adjustment (after matching: 23.7 months vs 16.9 months, respectively; before matching: 22.0 months vs 16.9 months, respectively). The median OS for the chemotherapy arm of KN021G/KN189/KN407 was 13.7 months after matching and 13.6 months before matching, both of which were comparable to the median OS of 14.9 months for the chemotherapy arm of CheckMate 227 Part 1A.

For PFS, the estimated HR based on the primary approach of pembrolizumab + chemotherapy vs nivolumab + ipilimumab was 0.53 after matching, similar to the before matching result which was 0.58. Based on the secondary approach, the estimated HR for PFS with pembrolizumab + chemotherapy vs nivolumab + ipilimumab was 0.55 after matching, similar to the before matching result, which was 0.62. Similar to the OS, the median PFS was higher among patients who received pembrolizumab + chemotherapy compared to those who received nivolumab + ipilimumab, regardless of adjustment (after matching: 10.7 months vs 5.0 months, respectively; before matching: 9.2 months vs 5.0 months, respectively). The median PFS for the chemotherapy arm of KN021G/KN189/KN407 was 4.9 months after matching and 4.9 months before matching, both of which were comparable to the median PFS of 5.5 months for the chemotherapy arm of CheckMate 227 Part 1A.

The results of the landmark analysis for OS and PFS at 6 months, 1 year and 2 years were in line with the base-case analysis, showing a higher OS and PFS for pembrolizumab + chemotherapy (Table 3). After matching, the landmark 2-year OS rate was 49.2% for pembrolizumab + chemotherapy vs 39.8% for nivolumab + ipilimumab, showing an absolute difference of approximately 10%. Similarly, pembrolizumab + chemotherapy demonstrated a higher landmark 2 years PFS rate of 28.0% vs 22.0% for nivolumab + ipilimumab after matching. The landmark analysis of the chemotherapy arms from KN021G/KN189/KN407 and CheckMate 227 Part 1A showed comparable OS and PFS at 6 months, 1 year and 2 years (Table S2). After matching, the landmark 2-year OS rate was 32.4% vs 33.3% for the chemotherapy arm from KN021G/KN189/KN407 and CheckMate 227 Part 1A, respectively, and the 2-year PFS rate was 4.7% vs 7.3%, respectively.

A comparison of the chemotherapy arms from KN021G/KN189/KN407 and CheckMate 227 Part 1A demonstrated similar results, suggesting the studies were highly comparable.

**Figure 1:**
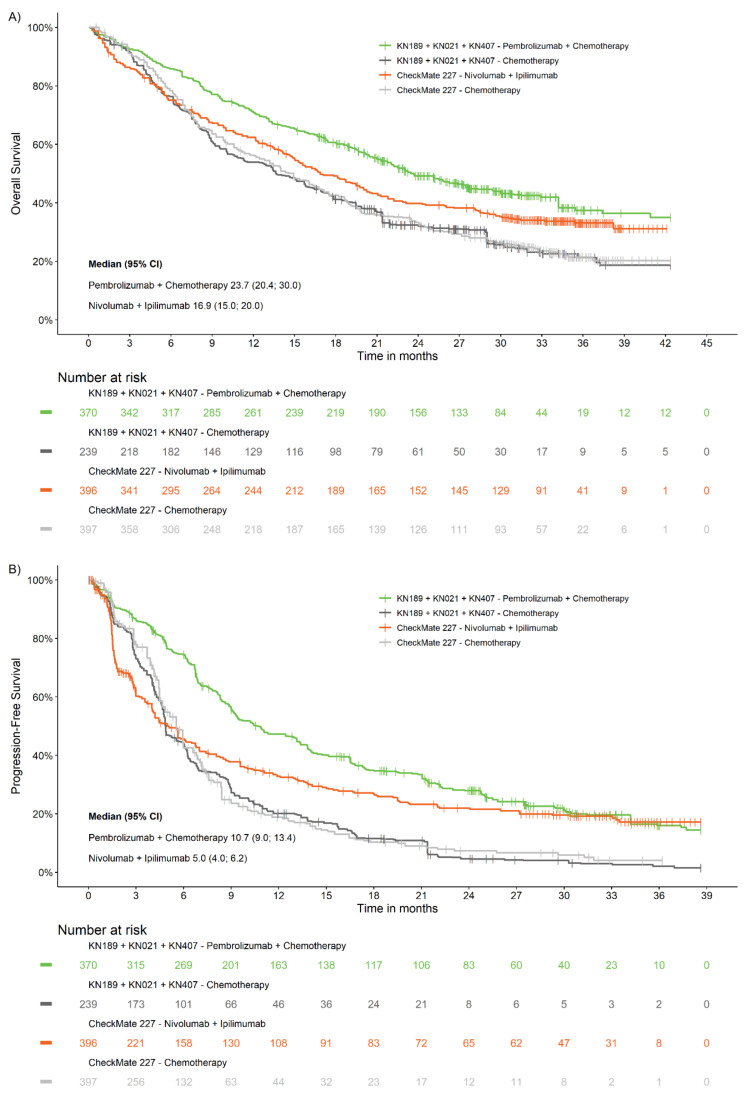
Kaplan–Meier curves after matching adjustment for (**A**) overall survival (**B**) progression-free survival.

The Kaplan–Meier plots of OS and PFS analyses for pembrolizumab + chemotherapy vs nivolumab + ipilimumab after matching are illustrated in Figure 1 (see Figure S1 for Kaplan–Meier plots before matching). Based on these plots, the curves for the pembrolizumab + chemotherapy and nivolumab + ipilimumab arms showed a separation for both OS and PFS endpoints. The curves for the chemotherapy arms from KN021G/KN189/KN407 and CheckMate 227 Part 1A were highly comparable in the OS and PFS Kaplan–Meier plots, after matching.

The proportion of patients with confirmed ORR after matching was 59.2% and 27.9% in the pembrolizumab + chemotherapy and chemotherapy arms, for a risk difference of 31.4 (95% CI 23.1‒39.7). The confirmed ORR after matching in the nivolumab + ipilimumab arm was 35.9% and 30.0% in the chemotherapy arm, for a risk difference of 5.9 (95% CI −0.6‒12.4). After matching, the risk ratio of pembrolizumab + chemotherapy vs nivolumab + ipilimumab was 1.8 (95% CI 1.3‒2.4; *p* value < 0.001). The risk difference was 25.5 (95% CI 15.0‒36.0; *p* value < 0.001).

### 3.3. Sensitivity Analyses and Sub-Group Analyses by PD-L1 Expression

**Figure 2:**
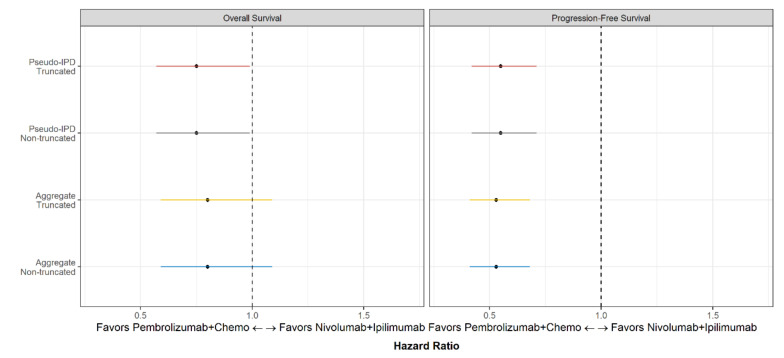
Forest plot of pembrolizumab + chemotherapy vs nivolumab + ipilimumab for overall survival and progression-free survival in patients with PD-L1 TPS ≥1%. Note: The results illustrated with the yellow lines refer to the base case analysis. IPD: individual patient data; PD-L1: Programmed cell death ligand 1; TPS: tumor proportion score.

Sensitivity analyses, conducted using the truncated and complete follow-up from each study (non-truncated), were consistent with the base case analysis between the two treatment options, showing a greater clinical benefit of pembrolizumab + chemotherapy compared with nivolumab + ipilimumab in the PD-L1 TPS ≥1% patient population after matching (Figure 2). The magnitude of the estimated treatment effect on OS and PFS appeared even more favorable towards pembrolizumab + chemotherapy compared with nivolumab + ipilimumab in the PD-L1 TPS 1–49% sub-group vs the PD-L1 TPS ≥50% sub-group, with an OS HR of 0.68 and 0.89, respectively, and a PFS HR of 0.46 and 0.56, respectively (Figure S2). A detailed breakdown of the sub-group analyses is available in Table S3.

## 4. Discussion

With the growing number of regulatory approved treatment regimens, it is increasingly important to understand the differences between these options to guide clinicians towards optimal treatment recommendations. Across the various clinical outcomes analyzed, pembrolizumab + chemotherapy appears to have a greater clinical benefit relative to nivolumab + ipilimumab in patients with recurrent/metastatic NSCLC with PD-L1 TPS ≥1%. Additionally, as clinical decision-making takes PD-L1 expression into account and subsets of patients may receive differing treatment choices accordingly, the sub-group analyses conducted here provide important results. The sub-group analyses were generally consistent with results in the overall PD L1 TPS ≥1% population, demonstrating the robust nature of the results and providing confidence that the results apply across a range of patients.

A future clinical trial examining the effectiveness of pembrolizumab + chemotherapy compared to nivolumab + ipilimumab would provide definitive and valuable answers. However, no such trial exists yet for advanced NSCLC patients with PD-L1 TPS ≥1% due to the expected large scale study requirements and the long time-frame required to achieve results. Further updated analyses, including evaluating longer-term OS, may help to consolidate and augment the analysis conducted here. The MAIC presented here provides clinically important results given the lack of head-to-head studies comparing pembrolizumab + chemotherapy vs nivolumab + ipilimumab. Given that both pembrolizumab + chemotherapy and nivolumab + ipilimumab are FDA-approved regimens for NSCLC patients with PD-L1 TPS ≥1%, these analyses are useful to inform physician decision-making and policy makers in this indication, when also considering other factors such as tolerability and cost of treatment.

The MAIC methodology represents an important strength of the study to generate comparative results. Standard ITC uses aggregate clinical trial data and therefore assumes a similar baseline distribution of effect modifiers across trials. Treatment comparisons may be subject to bias if this assumption is not met. Therefore, the MAIC methodology may be considered a more robust approach by matching on baseline characteristics the trial with IPD to the trial with aggregate data.

Despite the adoption of rigorous matching-adjusted criteria, there are several limitations to consider. First, the KEYNOTE and CheckMate trials have different sponsors. While key differences in the protocol of the studies and patient characteristics (outlined in Table 1) were accounted for, some subtle differences between study populations that the authors could not adjust for might remain. Additionally, while this study examined a wide range of baseline factors to mitigate any bias, and results were explored by PD-L1 expression sub-group, unreported or unmeasured differences in patient populations or study designs may still introduce bias into the analysis. Second, a higher percentage of patients who crossed over from the control arm to PD-L1/PD-1 within or outside of the trial was reported in the pooled KN021G/KN189/KN407 trials (53.5%) than CheckMate 227 Part 1A (43.1%). Anti-PD-1/PD-L1 inhibitors have been shown to improve OS in previously treated NSCLC patients with PD-L1 expression [25,26]. The matching of key baseline characteristics, and despite differences in cross-over, the OS Kaplan–Meier plots in the chemotherapy arms in the pooled KEYNOTE trials and CheckMate 227 Part 1A were very similar. Third, sub-group analyses were limited by data availability of PD-L1 TPS sub-group baseline data from CheckMate 227 Part 1A. Although there were differences in the way PD-L1 expression was evaluated in trials, studies have highlighted that the Dako22C3 assay (used in KN024/KN042) and Dako 28-8 assay (used in CheckMate 227 Part 1A) are highly concordant, therefore this is expected to have little impact on the results [27].

Adverse events (AEs) comparisons were not included due to potential biases in AE collection, reporting, and follow-up periods between studies. In KN189, ≥grade 3 AEs of any cause occurred in 67.2% of patients who received pembrolizumab + chemotherapy vs 65.8% of those who received placebo only [6]. In CheckMate 227 Part 1A, grade 3/4 treatment-related AEs were similar in the group that received nivolumab + ipilimumab and in the chemotherapy group (32.8% vs 36.0%) [9]. Although AEs were reported differently, the studies do not suggest a substantial difference in the overall incidence of AEs between the experimental and control arms. Therefore, the overall incidence of AEs between a combination of chemotherapy and immunotherapy vs nivolumab + ipilimumab are likely to be similar.

## 5. Conclusions

In conclusion, these MAIC results show that pembrolizumab + chemotherapy leads to a greater clinical benefit than nivolumab + ipilimumab in patients with PD‑L1 ≥1%.

## Figures and Tables

**Table 1:** Study characteristics for CheckMate 227 Part 1A, KEYNOTE-021 Cohort G, KEYNOTE-189 and KEYNOTE-407.

Category	CheckMate 227 Part 1A	KEYNOTE-021G	KEYNOTE-189	KEYNOTE-407
Study design and timeframe				
Study phase	III	II	III	III
Masking	Open-label	Open-label	Double-blind	Double-blind
Stratification factors	Tumor histology	PD-L1 TPS	PD-L1 TPS, platinum-based drug and smoking history	Paclitaxel vs nab-paclitaxel, PD-L1 status and region of enrollment
Patient enrollment period	August 2015–November 2016	November 2014–January 2016	February 2016–March 2017	August 2016–December 2017
Median duration of follow-up	Not reported (Minimum follow-up duration 29.3 months)	31.0 months (range: 0.8–55.4)	18.8 months (range: 0.2–38.8)	14.3 months (range: 0.1–31.3)
Maximum permitted duration of immunotherapy	2 years	2 years	2 years	2 years
Key inclusion criteria				
Minimum age (years)	18	18	18	18
ECOG performance score	≤1	≤1	≤1	≤1
Disease stage	IV or recurrent	IIIb or IV	IV	IV
EGFR mutation and/or ALK rearrangement positive patients excluded	Yes	Yes	Yes	Not applicable ^a^
Cross-over				
Cross-over allowed within trial	Not permitted	Permitted	Permitted	Permitted
Condition for within trial cross-over	Not applicable	Progressive disease defined by RECIST v1.1, investigator assessed	Progression verified by blinded, independent central radiologic review	Progression verified by blinded, independent central radiologic review
Cross-over from	Not applicable	Chemotherapy	Chemotherapy	Chemotherapy
Cross-over to	Not applicable	Pembrolizumab monotherapy	Pembrolizumab monotherapy	Pembrolizumab monotherapy
Number of patients crossed over to PD1 inhibitor within trial	Not applicable	28/63 (44.4%)	84/206 (40.8%)	114/281 (40.6%)
Subsequent immunotherapy or PD-1/PD-L1-directed therapy in the control group (within trial + outside of trial crossover)	171/397 (43.1%)	43/63 (68.3%)	113/206 (54.9%)	138/281 (49.1%)

^a^ Routine testing for EGFR/ALK is not recommended for squamous NSCLC by ESMO and NCCN guidelines, except in selected patients with squamous NSCLC if they are never smokers, small biopsy specimens were used for testing, or mixed histology was reported. ALK: anaplastic lymphoma kinase; ECOG: Eastern Cooperative Oncology Group; EGFR: epidermal growth factor receptor; ESMO: European Society for Medical Oncology; NCCN: National Comprehensive Cancer Network; NSCLC: non-small cell lung cancer; ORR: objective response rate; OS: overall survival; PD-1: programmed cell death protein 1; PD-L1: programmed death-ligand 1; PFS: progression-free survival; RECIST: Response evaluation criteria in solid tumors; TPS: tumor progression score.

**Table 2:** Baseline characteristics before and after matching.

Category	Nivolumab + Ipilimumab and Chemotherapy Arms CheckMate 227 Part 1A (*n* = 793)	Pembrolizumab + Chemotherapy and Chemotherapy Arms KN021G/KN189/KN407	
		Before Matching (*n* = 816)	After Matching (*n* = 456 ^a^)
Age Group (years)			
≤64	51.20	46.69	51.20
65–74	38.59	42.77	38.59
≥75	10.21	10.54	10.21
Sex			
Male	64.94	66.30	64.94
Female	35.06	33.70	35.06
Region			
North America	11.98	25.86	11.98
Europe	50.44	48.65	50.44
Asia	20.43	8.46	20.43
Rest of World	17.15	17.03	17.15
Smoking Status ^b^			
Current/Former	86.30	88.85	86.30
Never	13.70	11.15	13.70
ECOG Status			
0	33.92	37.25	33.92
1	65.45	62.25	65.45
Other score or missing data	0.63	0.49	0.63
Histology			
Non-Squamous	70.62	56.74	70.62
Squamous	29.38	43.26	29.38
Metastases			
Bone	26.23	32.97	26.23
CNS	10.21	11.89	10.21
Liver	19.67	18.14	19.67
PD-L1 TPS Group			
1–49	49.94	53.19	49.94
≥50	50.06	46.81	50.06

The results are presented in percentages. ^a^: Effective sample size computed as the square of the summed weights divided by the sum of the squared weights. ^b^: Due to missing values in CheckMate 227 Part 1A, percentages and total *n* were recalculated based on available information after excluding missing values, matching was then based on recalculated percentages. CNS: Central Nervous System; ECOG: Eastern Cooperative Oncology Group; KN021G: KEYNOTE-021 Cohort G; KN189: KEYNOTE-189; KN407: KEYNOTE-407; PD-L1: Programmed cell death ligand 1; TPS: tumor proportion score.

**Table 3:** Overall survival and progression-free survival in patients with PD-L1 TPS ≥1% after matching.

Category	Outcomes	
	Overall Survival	Progression-Free Survival
ITC HR (95% CI), *p* value ^a^		
Primary approach ^b^	0.80 (0.59, 1.09), 0.152	0.53 (0.41, 0.68), <0.001
Secondary approach ^c^	0.75 (0.57, 0.99), 0.039	0.55 (0.42, 0.71), <0.001
Number of events, (%) ^d^		
Pembrolizumab + chemotherapy	206 (55.9)	277 (75.1)
Nivolumab + ipilimumab	259 (65.4)	289 (73.0)
KN021G/KN189/KN407: Chemotherapy ^e^	174 (72.6)	222 (92.8)
Checkmate 227: Chemotherapy ^e^	299 (75.3)	286 (72.0)
Median Months, (95% CI)		
Pembrolizumab + chemotherapy	23.7 (20.4; 30.0)	10.7 (9.0; 13.4)
Nivolumab + ipilimumab	16.9 (15.0; 20.0)	5.0 (4.0; 6.2)
KN021G/KN189/KN407: Chemotherapy ^e^	13.7 (10.1; 17.8)	4.9 (4.6; 6.1)
Checkmate 227: Chemotherapy ^e^	14.9 (12.5; 16.8)	5.5 (4.8; 5.9)
Landmark rate (%)–Pembrolizumab + chemotherapy vs nivolumab + ipilimumab		
6-month	85.8 vs. 75.2	74.5 vs. 45.6
1-year	71.0 vs. 62.4	47.2 vs. 32.8
2-year	49.2 vs. 39.8	28.0 vs. 22.0

^a^: Two-sided *p* value calculated from the test statistic associated with the ITC estimate and its standard error. ^b^: Calculated using aggregate data published in the literature for nivolumab + ipilimumab. Bucher methodology using separate study results (estimate and its standard error) with a common control arm. ^c^: Calculated using pseudo-IPD from CheckMate227 Part 1A using a Cox regression model. ^d^: For pembrolizumab + chemotherapy and KN021G/KN189/KN407 chemotherapy, percentage was calculated based on sample size after matching, computed as the sum of the weights. ^e^: Platinum-doublet chemotherapy for KN021G/KN0189/KN407 and CheckMate227 Part 1A. CI: confidence interval; ITC: indirect treatment comparison; HR: hazard ratio; ITT: Intention-to-Treat; KN021: KEYNOTE-021 Cohort G; KN189: KEYNOTE-189; KN407: KEYNOTE-407; PD-L1: Programmed cell death ligand 1; TPS: tumor proportion score.

## Supplementary Materials

The following are available online at https://www.mdpi.com/2072-6694/12/12/3648/s1, Table S1: Overall survival and progression-free survival in patients with PD-L1 TPS ≥1% before matching; Table S2: Landmark analysis of overall survival and progression-free survival for chemotherapy arms from KN021G/KN189/KN407 and CheckMate 227 Part 1a before and after matching; Table S3: Pembrolizumab vs nivolumab + ipilimumab for overall survival and progression-free survival in patients with PD-L1 TPS ≥1%, TPS ≥50% and TPS 1–49%; Figure S1: Kaplan–Meier curves before matching adjustment for (**a**) overall survival (**b**) progression-free survival; Figure S2: Forest plot of pembrolizumab + chemotherapy vs nivolumab + ipilimumab for overall survival and progression-free survival in patients with PD-L1 TPS 1–49% and ≥50%
