# Supplementary material for: A Matching-Adjusted Indirect Comparison of Pembrolizumab + Chemotherapy vs. Nivolumab + Ipilimumab as First-Line Therapies in Patients with PD-L1 TPS ≥1% Metastatic NSCLC
Source: Cancers (Basel). 2020 Dec 4;12(12):3648. doi: 10.3390/cancers12123648 (PMC7762014; doi:10.3390/cancers12123648)
Supplement: Supplementary file 1 [file cancers-12-03648-s001.pdf]

cancers

MDPI

Supplementary materials: A Matching-Adjusted Indirect Comparison of Pembrolizumab + Chemotherapy vs Nivolumab + Ipilimumab as First-Line Therapies in Patients with PD-L1 TPS ≥1% Metastatic NSCLC

Balazs Halmos, Thomas Burke, Chrysostomos Kalyvas, Ralph Insinga, Kristel Vandormael, Andrew Frederickson and Bilal Piperdi

Table S1. Overall survival and progression-free survival in patients with PD-L1 TPS≥1% before matching.

	Outcomes	
	Overall Survival	Progression-Free Survival
**ITC HR (95% CI), p-value[a]**		
Primary approach[b]	0.84 (0.63, 1.11), 0.218	0.58 (0.46, 0.74), <0.001
Secondary approach[c]	0.82 (0.64, 1.04), 0.102	0.62 (0.49, 0.77), <0.001
**Number of events, (%)**		
Pembrolizumab+chemotherapy	273 (57.6)	356 (75.1)
Nivolumab+ipilimumab	259 (65.4)	289 (73.0)
KN189/407/021G: Chemotherapy[d]	241 (70.5)	310 (90.6)
Checkmate 227: Chemotherapy[d]	299 (75.3)	286 (72.0)
**Median Months, (95% CI)**		
Pembrolizumab+chemotherapy	22.0 (19.7; 25.2)	9.2 (8.3; 10.9)
Nivolumab+ipilimumab	16.9 (15.0; 20.0)	5.0 (4.0; 6.2)
KN189/407/021G: Chemotherapy[d]	13.6 (10.9; 15.7)	4.9 (4.6; 6.0)
Checkmate 227: Chemotherapy[d]	14.9 (12.5; 16.8)	5.5 (4.8; 5.9)
**Landmark rate (%) – Pembrolizumab+chemotherapy vs. Nivolumab+ipilimumab**		
6-month	85.4 vs 75.2	70.2 vs 45.6
1-year	69.3 vs 62.4	43.5 vs 32.8
2-year	46.6 vs 39.8	26.7 vs 22.0

a: Two-sided p-value calculated from the test statistic associated with the ITC estimate and its standard error

b: Calculated using aggregate data published in the literature for nivolumab+ipilimumab. Bucher methodology using separate study results (estimate and its standard error) with a common control arm

c: Calculated using pseudo-IPD from CheckMate227 Part 1A using a Cox regression model

d: Platinum-doublet chemotherapy for KN021G/KN0189/KN407 and CheckMate227 Part 1A

CI: confidence interval; ITC: indirect treatment comparison; HR: hazard ratio; ITT: Intention-to-Treat; KN021: KEYNOTE-021 Cohort G; KN189: KEYNOTE-189; KN407: KEYNOTE-407; PD-L1: Programmed cell death ligand 1; TPS: tumor proportion score.

Table S2. Landmark analysis of overall survival and progression-free survival for chemotherapy arms from KN021G/KN189/KN407 and CheckMate 227 Part 1a before and after matching.

	**Landmark rate 6 month,** %	**Landmark rate 1-year ,** %	**Landmark rate 2-year,** %
Overall survival			
Before Matching	76.5 vs 78.4	53.3 vs 56.3	34.3 vs 33.3
After Matching	76.4 vs 78.4	53.9 vs 56.3	32.4 vs 33.3
Progression-free survival			
Before Matching	44.6 vs 42.6	20.3 vs 18.8	8.0 vs 7.3
After Matching	44.1 vs 42.6	20.2 vs 18.8	4.7 vs 7.3

KN021G: KEYNOTE-021 Cohort G; KN189: KEYNOTE-189; KN407: KEYNOTE-407.

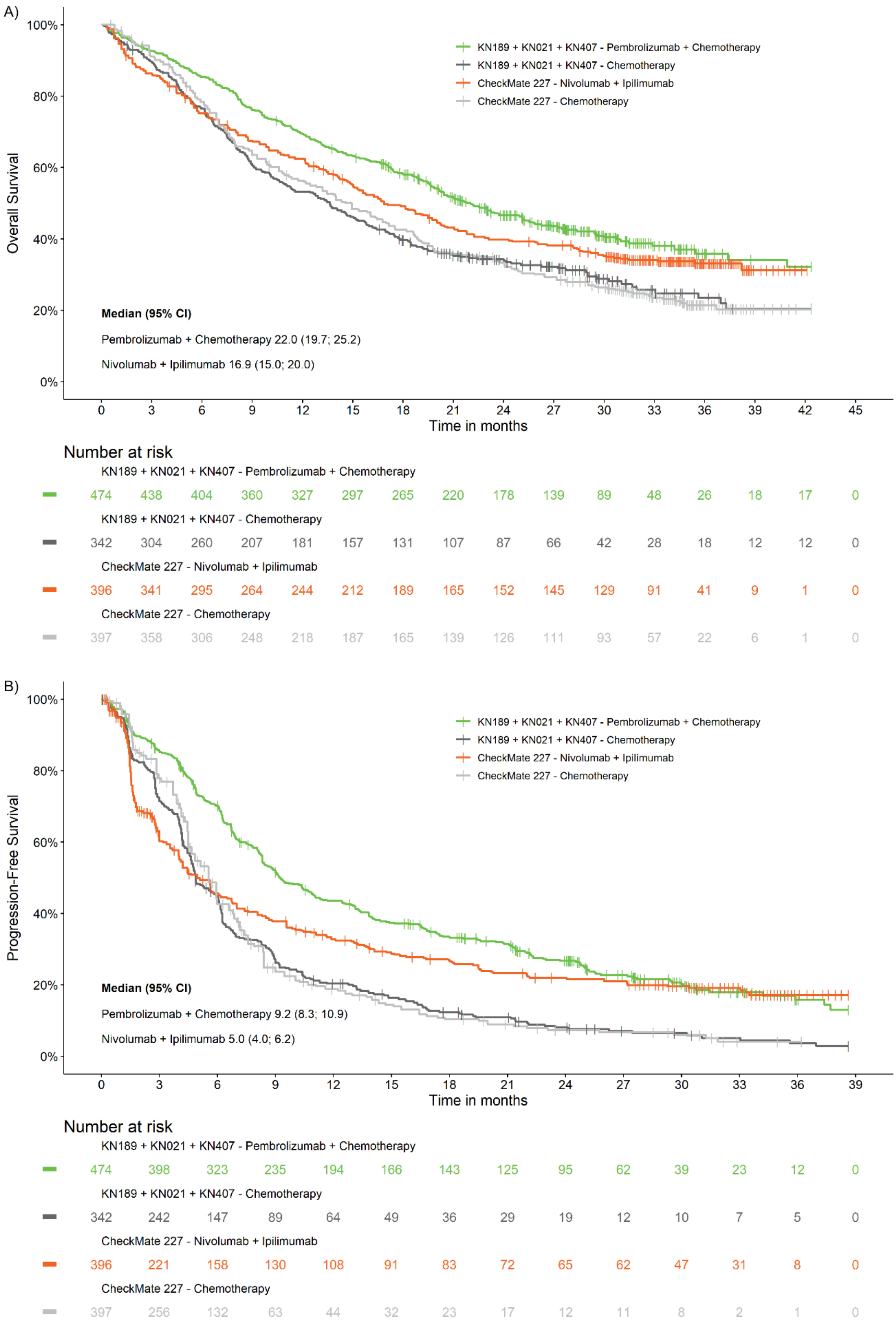

Figure S1. Kaplan–Meier curves before matching adjustment for a) overall survival b) progression-free survival.

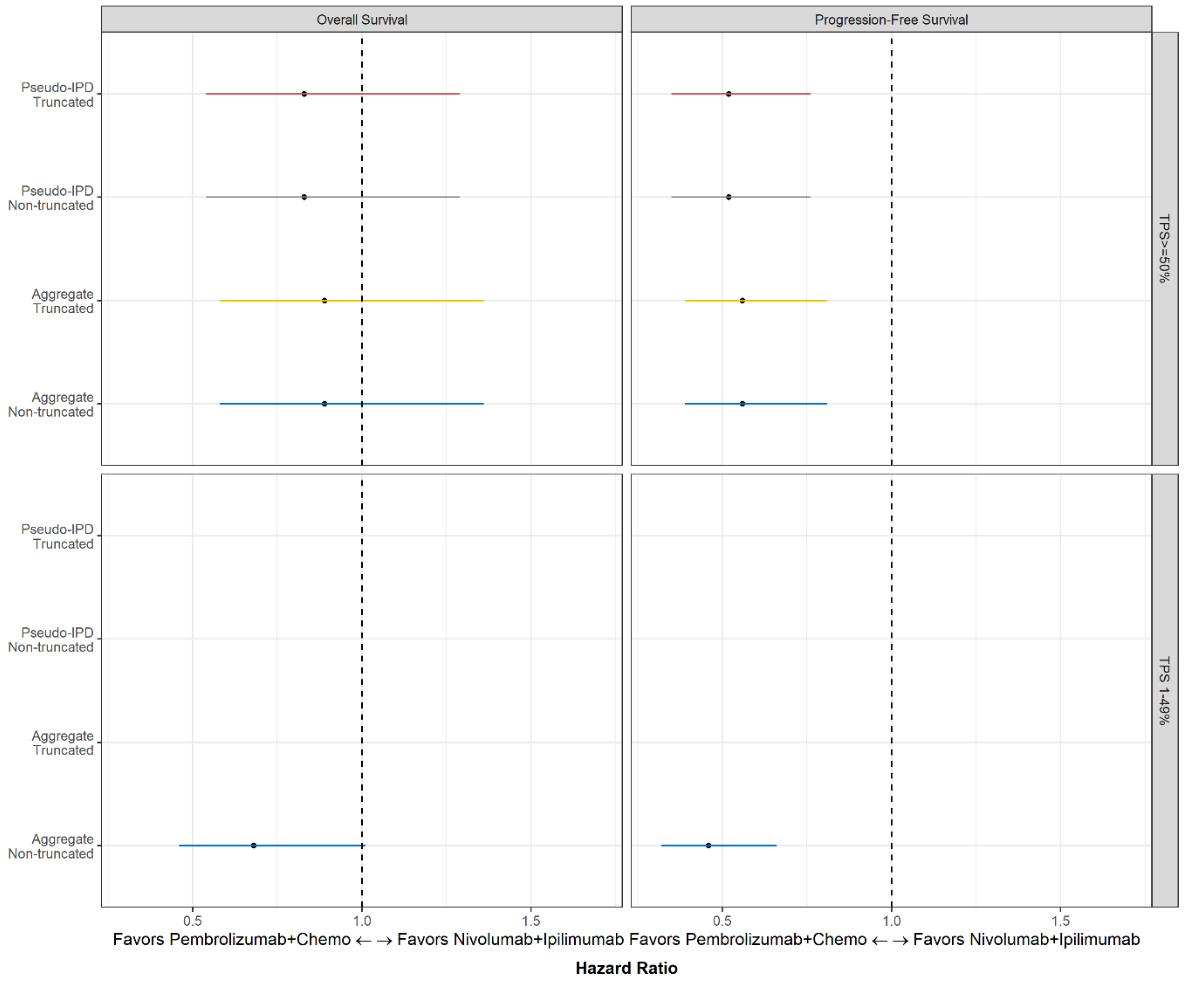

Figure S2. Forest plot of pembrolizumab+chemotherapy versus nivolumab+ipilimumab for overall survival and progression-free survival in patients with PD-L1 TPS 1–49% and ≥50%[a].

[a]: The results illustrated with the yellow lines refer to the base case analysis.

IPD: individual patient data; PD-L1: Programmed cell death ligand 1; TPS: tumor proportion score.

Table S3. Pembrolizumab versus nivolumab+ipilimumab for overall survival and progression-free survival in patients with PD-L1 TPS≥1%, TPS≥50% and TPS 1–49%.

	Hazard Ratio (95% CI)	
	Before Matching	After Matching
**Overall Survival, ≥1%**		
Aggregate, truncated[a]	0.84 (0.63, 1.11)	0.80 (0.59, 1.09)
Pseudo IPD, truncated	0.82 (0.64, 1.04)	0.75 (0.57, 0.99)

Aggregate, non-truncated	0.84 (0.64, 1.11)	0.80 (0.59, 1.09)
Pseudo IPD, non-truncated	0.82 (0.64, 1.04)	0.75 (0.57, 0.99)
**Progression-Free Survival (BICR), ≥1%**		
Aggregate, truncated[a]	0.58 (0.46, 0.74)	0.53 (0.41, 0.68)
Pseudo IPD, truncated	0.62 (0.49, 0.77)	0.55 (0.42, 0.71)
Aggregate, non-truncated	0.58 (0.46, 0.74)	0.53 (0.41, 0.68)
Pseudo IPD, non-truncated	0.62 (0.49, 0.78)	0.55 (0.42, 0.71)
**Overall Survival, ≥50%**		
Aggregate, truncated[a]	0.90 (0.63, 1.29)	0.89 (0.58, 1.36)
Pseudo IPD, truncated	0.87 (0.60, 1.24)	0.83 (0.54, 1.29)
Aggregate, non-truncated	0.90 (0.63, 1.29)	0.89 (0.58, 1.36)
Pseudo IPD, non-truncated	0.87 (0.60, 1.24)	0.83 (0.54, 1.29)
**Progression-Free Survival (BICR), ≥50%**		
Aggregate, truncated[a]	0.63 (0.45, 0.88)	0.56 (0.39, 0.81)
Pseudo IPD, truncated	0.63 (0.45, 0.89)	0.52 (0.35, 0.76)
Aggregate, non-truncated	0.63 (0.45, 0.88)	0.56 (0.39, 0.81)
Pseudo IPD, non-truncated	0.63 (0.45, 0.89)	0.52 (0.35, 0.76)
**Overall Survival, 1-49%**		
Aggregate, non-truncated	0.71 (0.50, 1.01)	0.68 (0.46, 1.01)
**Progression-Free Survival (BICR), 1-49%**		
Aggregate, non-truncated	0.49 (0.35, 0.69)	0.46 (0.32, 0.66)

[a]: The aggregate/truncated analysis corresponds to the base case analysis and is included for comparison purposes.

BICR: blinded independent committee review; CI: confidence intervals; IPD: individual patient data; PD-L1: Programmed cell death ligand 1; TPS: tumor proportion score.
